# Medical student education through flipped learning and virtual rotations in radiation oncology during the COVID-19 pandemic: a cross sectional research

**DOI:** 10.1186/s13014-021-01927-x

**Published:** 2021-10-16

**Authors:** Tae Hyung Kim, Jin Sung Kim, Hong In Yoon, Joongyo Lee, Jason Joon Bock Lee, Hwa Kyung Byun, Yeona Cho, Yong Bae Kim, Ik Jae Lee, Kyung Hwan Kim, Jee Suk Chang

**Affiliations:** 1 Department of Radiation Oncology, Yonsei University College of Medicine, 50-1 Yonsei-ro, Seodaemun-gu, Seoul, 03722 Republic of Korea; 2 Department of Radiation Oncology, Nowon Eulji Medical Center, Eulji University School of Medicine, Seoul, Republic of Korea; 3 Department of Radiation Oncology, Kangbuk Samsung Hospital, Sungkyunkwan University School of Medicine, Seoul, Republic of Korea; 4 Gangnam Severance Hospital, Seoul, Republic of Korea

**Keywords:** COVID-19, Medical education, Flipped learning

## Abstract

**Background:**

The COVID-19 pandemic has stripped many medical students worldwide of their right to quality education. In response, we developed hybrid courses involving aspects of both online and in-person teaching for radiation oncology medical student clerkship.

**Methods:**

We entitled students to customize their own rotation schedule using Google Forms and developed a flipped learning online class, which consisted of at least one video clip on basic knowledge of radiation oncology per day (yonsei-radonc.com). Students were instructed to watch online videos before the next day’s discussion session. Required components of the medical education program (e.g., target drawing, site visits to treatment facilities) were also prepared and conducted in accordance with the appropriate level of social distancing measures. Finally, we conducted questionnaire surveys after the completion of the week-long course and clerkship.

**Results:**

From March to June 2020, 110 fourth-year medical students undertook a clinical module in our 1-week radiation oncology program course. Each day, students completed the flipped learning prior to meeting with the educator and then participated in the online discussion session and conference. All activities were well performed as scheduled. Students’ motivation was high, as was their overall satisfaction with the course. The students were satisfied with the online contents, flipped learning strategy, and instructors.

**Conclusions:**

We successfully integrated open and virtual educational platforms to improve access to and satisfaction with student clerkship. In the future “new normal,” minimized face-to-face learning interactions, such as flipped learning, should be actively utilized for medical and other students’ education.

**Supplementary Information:**

The online version contains supplementary material available at 10.1186/s13014-021-01927-x.

## Background

The COVID-19 pandemic has resulted in far-reaching consequences; the closure of classrooms, in particular, necessitated development of alternative methods of delivering education. Professionals in medical education worldwide have explored different methods of modality toward ensuring that students continue to receive quality education.

**Fig. 1:**
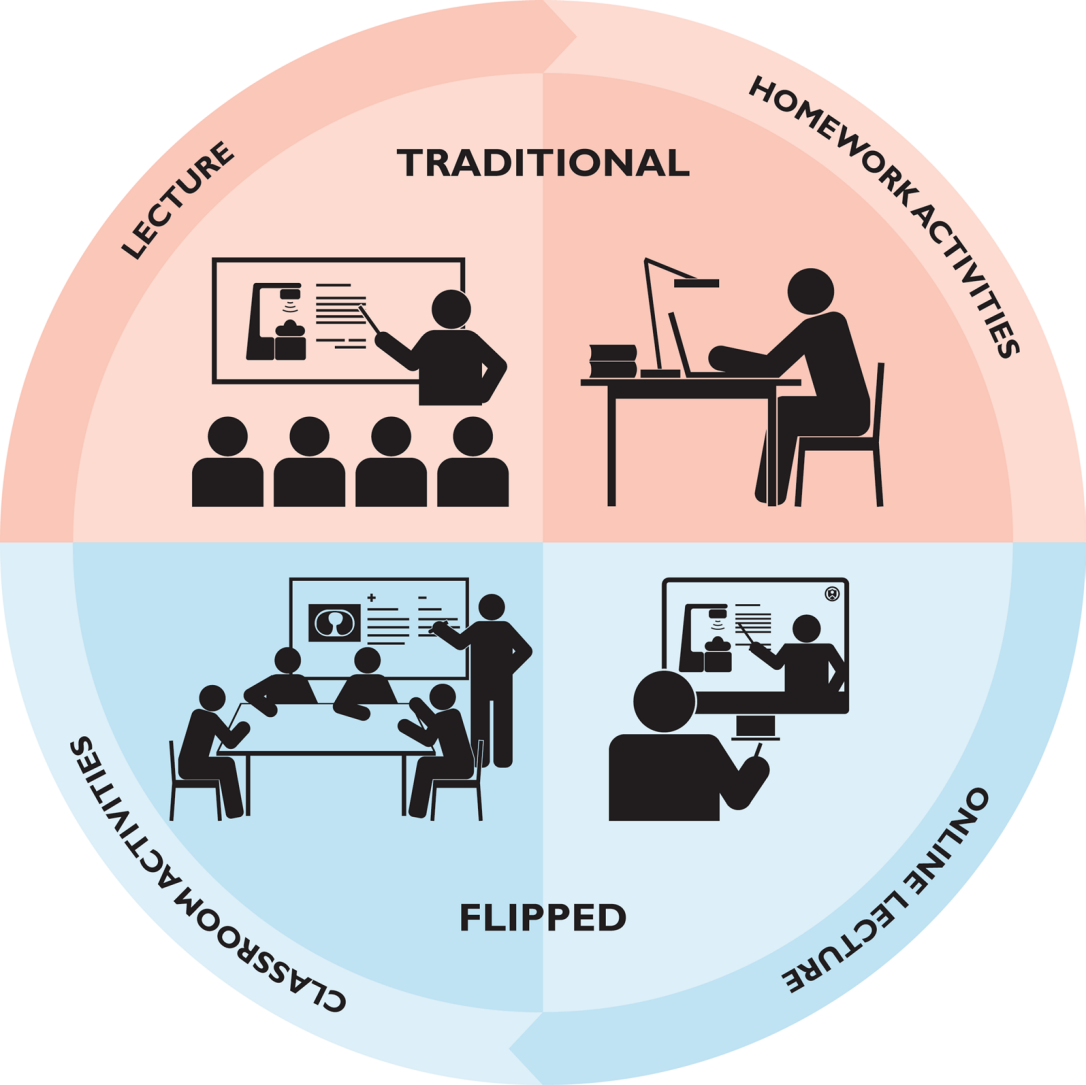
Flipped learning versus traditional learning. *Note*: The upper row shows traditional learning consisting of lectures conducted in person and homework activity. The lower row shows flipped learning consisting of lectures conducted online and classroom activities

Flipped learning is a teaching strategy that reverses traditional learning by delivering core content outside of the classroom, and shifts activities more traditionally thought of as homework, into the classroom (Fig. 1) [1]. Lectures have rapidly been developed to be delivered online via platforms such as ZOOM®, WebEx®, and Teams®, with such technologically enhanced approaches already being proven to have high levels of engagement with medical students [2]. Amid pandemic-related public health recommendations, education for medical students has quickly and effectively transitioned to virtual platforms. In this circumstance, radiation oncology is particularly well positioned to create and innovate virtual curricula for medical students [3].

In response to the COVID-19 crisis, we created a hybrid program comprising virtual and online parts for a 1-week radiation oncology rotation at Yonsei University College of Medicine. Feedback from and the experience of creating and implementing this novel program course will shape the education to be provided in the future. Moreover, the development of virtual education platforms can establish a unique niche within medical education.

## Methods

### Students’ customized schedule

According to the academic curriculum of Yonsei University College of Medicine, fourth-year medical students undertake a clinical module in radiation oncology. Our 1-week program course included 110 students divided into 16 groups. The course opened on a Sunday evening; students were provided with online links for the flipped learning website and an online questionnaire (Additional file 1: Fig. 1) that they were instructed to use for creating their own 1-week schedule. The online questionnaire had two categories. The first was on the required education curriculum and the second one was on the elective education curriculum that included various categories of available programs. Students had to choose an education location (either “Yonsei Cancer Center” or “Gangnam Severance Hospital”), more than one session in the outpatient clinic of a professor (7 selectable options for professor), more than two multi-disciplinary discussions (7 selectable options), more than one session in a multi-disciplinary team clinic (5 selectable options), and more than two actual radiotherapy sessions (including target/plan confirmation time with a professor, vaginal brachytherapy, and prostate permanent brachytherapy). Students could make their own 1-week schedule because they wanted to train in different outpatient clinics, multi-disciplinary team clinics, and actual radiotherapy session according to cancer subtype. Table 1 shows an example of a customized schedule for one student.

**Table 1:** Example Schedule for Students

Days	Monday	Tuesday	Wednesday	Thursday	Friday
8:00–9:00	(R) Introduction Safety instruction	(R) Discussion for lecture	(E) Attend prostate brachytherapy operation room	(R) Discussion for lecture	(E) Attend multi-disciplinary discussion
	Fellow	Fellow		Fellow	Professor
9:00–11:00	(E) Outpatient clinic	(R) Target drawing		(E) Attend target/plan confirmation time	(R) Discussion for lecture
	Professor	Fellow	Professor	Professor	Fellow
11:00–13:00	(R) Tour to treatment room and simulation room	(E) Multi-disciplinary discussion	(R) Visit carbon-ion therapy construction site	(E) Attend Cervix brachytherapy	(R) Wrap-up and discussion
	Resident	Professor	Resident	Resident/fellow	Professor
13:00–16:00	(E) Self-study for next day	(E) Self-study for next day	(E) Attend target/plan confirmation time	(E) Self-study for next day	
	Student	Student	Professor	Student	

*(R)* Required curriculum, *(E)* Elective curriculum

### Flipped learning website

For the students’ flipped learning, we created a website in which core topics on basic knowledge of radiation oncology, as well as basic and advanced techniques in radiotherapy, could be chosen. We uploaded selected lectures delivered in videos lasting 10–20 min. We prepared two program courses for each day (total of 10 lectures). Each day of the program was designed to enhance students’ understanding of radiation oncology (Table 2). Students were instructed to watch the online videos before the next day’s discussion session.

**Table 2:** Lectures delivered online

Days	Sunday	Monday	Tuesday	Wednesday	Thursday	Friday
Topic 1	Welcome address	Understanding RT process	How to target drawing	Particle therapy	Intensity-modulated RT	Future of radiation oncology
Topic 2	Introduction of Yonsei Rad Onc	Definition of target and OAR	Understanding DVH	Carbon-ion therapy	Prostate brachytherapy	Wrap-up
Instructor	YB Kim	JS Kim	JS Chang	WS Koom & HI Yoon	JH Cho & YA Cho	JS Chang

### Online conferences

**Fig. 2:**
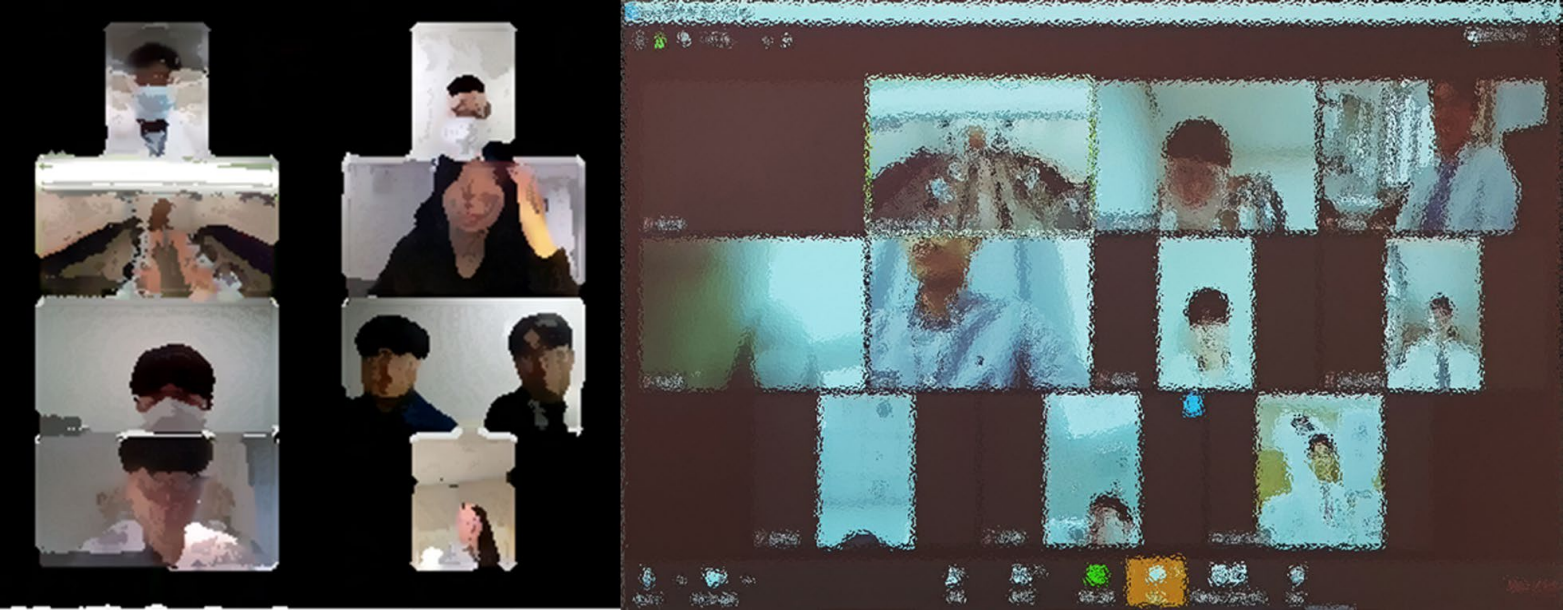
Internal conferences conducted online

Video conferencing equipment (Logitech® Meetup) was installed in a conference room. Before the COVID-19 pandemic, face-to-face internal conferences were held every morning: presentation of new patients (Monday), textbook review by resident (Tuesday), case conference (Wednesday), journal conference (Thursday), and biology/physics lecture (Friday). However, after the COVID-19 outbreak, the morning conferences began to be conducted online, in which students were instructed to participate and strongly encouraged to interact with educators (Fig. 2). The students participated in online conferences via ZOOM® or offline conferences in accordance with the appropriate social distancing measures. After the internal conferences, students participated in the discussion session with clinical fellows on the topic covered on the previous day.

### Required education program

Required education program components (e.g., target drawing, site visits to carbon–ion treatment facility construction site and treatment rooms at lunchtime) were also prepared and conducted in accordance with the appropriate social distancing measures.

Before the social distancing measures were strengthened, a program was planned to identify one patient in an outpatient clinic to perform the physical examination and history taking in person, and to observe the patient’s radiotherapy process. However, after the social distancing measures were strengthened because of the COVID-19 pandemic, medical school recommendations no longer allowed to meet patients in person and perform such processes. The Korean government is currently attempting to implement telemedicine, but it is not legally permissible yet. Since they were not allowed to meet patients online for counseling or medical treatment, students had no choice but to observe professors treating patients in outpatient clinics. Even in outpatient clinics, both doctors and patients were wearing masks, and conducting sessions with a plastic screen between them. Students were only allowed to observe the process from a distance.

A target drawing session was designed for all students with the same patient. Before contouring, the students were taught the concept of hippocampal-sparing whole brain radiotherapy. A 70-year-old male patient with solitary brain metastasis from small cell lung cancer received hippocampal-sparing whole brain radiotherapy. The students contoured the right and left hippocampus, gross tumor on the brain, and whole brain. After contouring, students were shown the actual target drawn by the radiation oncologist and actual radiotherapy plan received by the patient; these were subsequently compared with the students’ output.

The construction of a heavy carbon–ion treatment facility at Yonsei University College of Medicine is underway. All students visited the construction site where they attended a lecture on carbon–ion treatment, Bragg Peak of heavy ion, and the advantages and disadvantages of carbon treatment. In addition, all students experienced the operation of the computed tomography (CT) simulation room and linear accelerator and robotic intensity-modulated radiotherapy treatment room. In the CT simulation room, students who wanted to experience actual immobilization during CT simulation and thermoplastic immobilization device (called “S-plate”) were accommodated as appropriate. In the treatment room, the operation of a treatment machine was demonstrated in the absence of actual patients.

### Post-program survey

At the end of all programs of the academic curriculum, the College of Medicine typically conducts students’ surveys on both subject-specific recognition and overall satisfaction, with items being rated on a scale of 1 to 5. The subject-specific recognition survey consists of 12 items. Apart from the university surveys, departments may ask students for feedback. For the present study, online questionnaires prepared by our own department were sent out at the end of each rotation (16 weeks), and each category had either a 1-to-10 scoring system (1 = *worst* to 10 = *best*) or 1-to-5 scoring system (1 = *very poor*, 5 = *very good*). The categories included the skill and responsiveness of the instructor, quality of the webpage and video lecture, and overall satisfaction with the course content. The survey also included four free-response questions (Additional file 2: Fig. 2).

### Post-program student evaluation

Oral tests were conducted after all the programs were over. The oral test was conducted by a professor, a clinical fellow, and a student, one by one. The students’ understanding of the program was evaluated based on their answers to the oral test. Questions from the oral test were presented based on online lectures, contents of the online conference by residents, and the information discussed with the clinical fellow during the offline conference.

Students’ grades were comprehensively evaluated based on oral test scores, attendance scores, and attitude scores. Subsequently, the fourth-year medical students were graded as either “pass” or “fail”, and outstanding students received a “pass with honor” degree. Students who failed the initial program received a re-training program.

## Results

### Overall curriculum progress

**Fig. 3:**
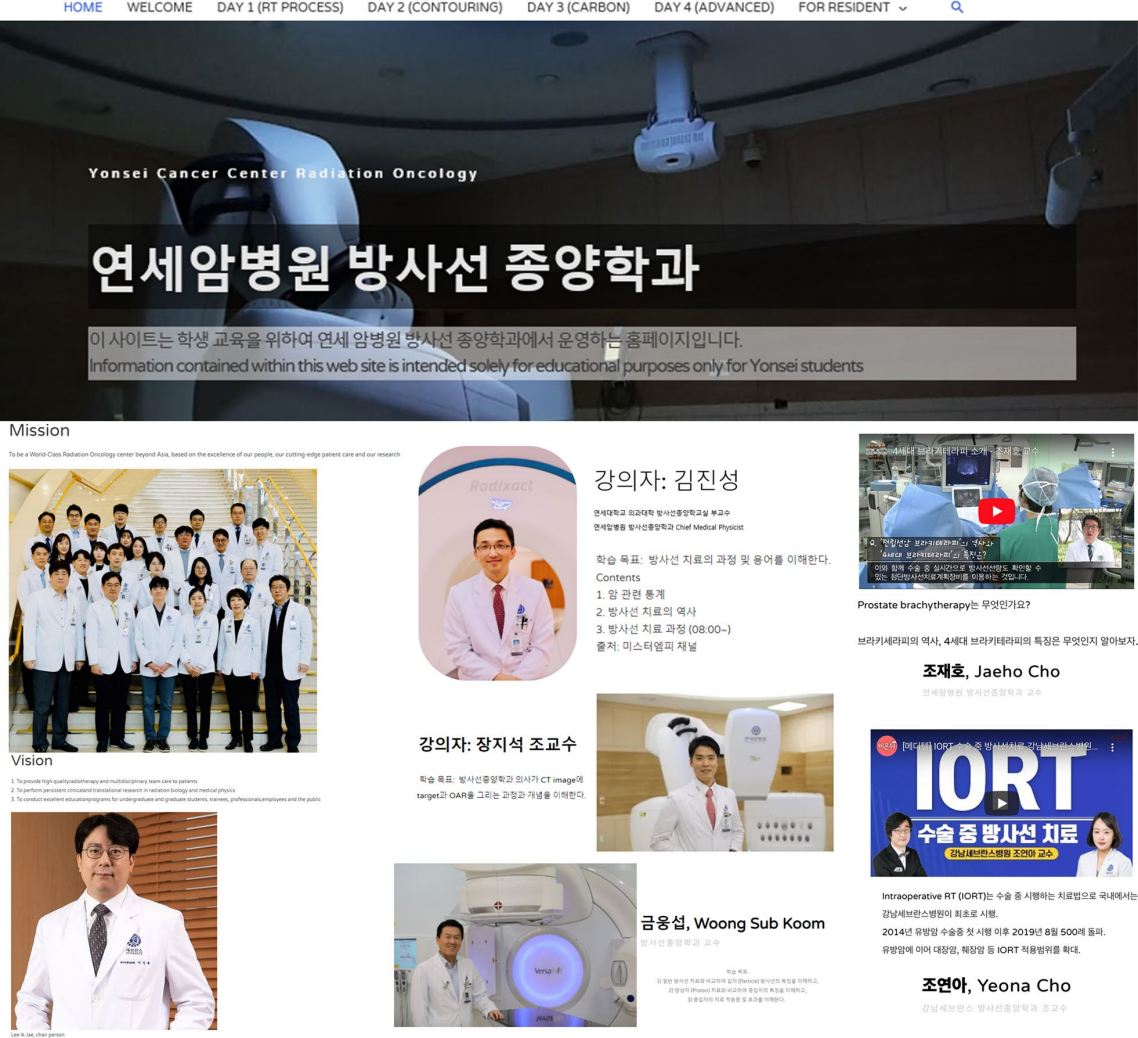
Flipped learning class (yonsei-radonc.com)

We successfully developed a flipped learning class (yonsei-radonc.com, Fig. 3). All students could utilize the website designed for flipped learning. All activities were performed as scheduled for all students, even with the 4-week break imposed after the COVID-19 outbreak.

**Fig. 4:**
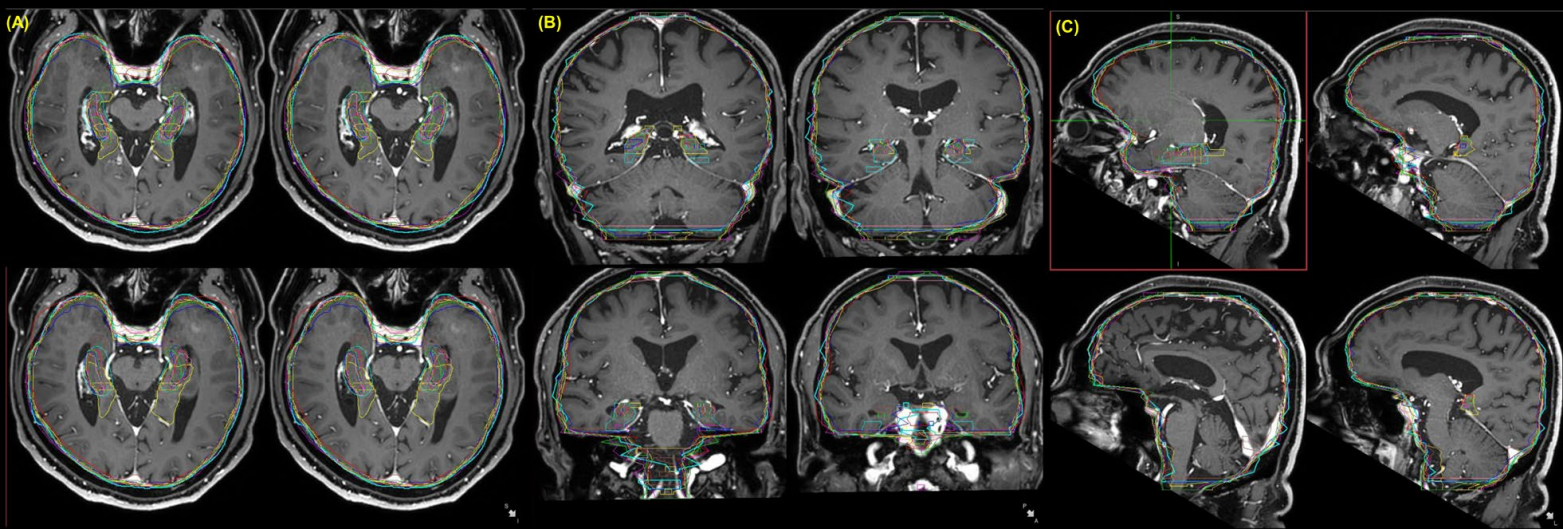
Target volume variation in students’ contouring

Figure 4 shows the target volume variation in students’ contouring. Most students could delineate brain metastasis well but showed substantial differences in terms of the contour of the hippocampus and brain structures. We showed the actual target volume used for whole brain radiotherapy to students after their contouring session and explained the consequences of therapy.

### Survey results

**Fig. 5:**
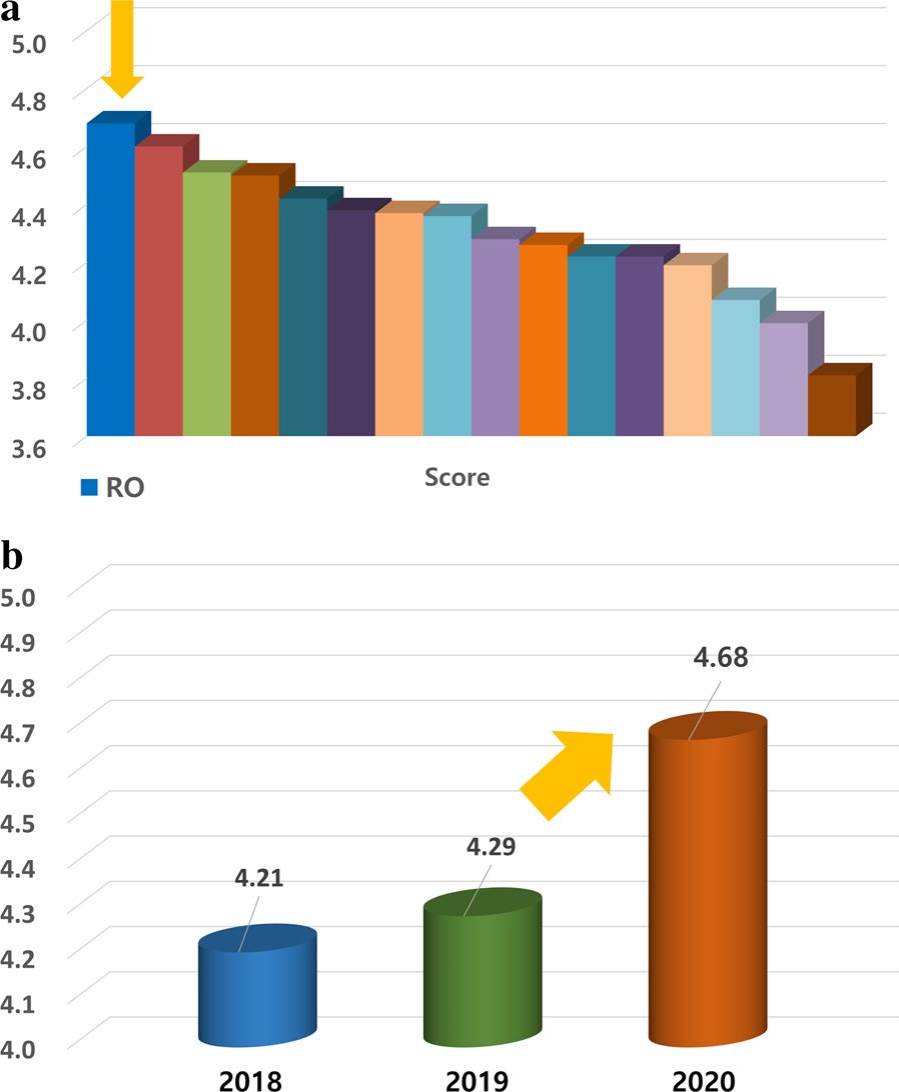
Survey results by school. **A** Overall satisfaction score in 16 departments. **B** Score changes for radiation oncology over 3 years

Figure 5 shows the results of the survey conducted by the school. Our department was ranked first among 16 departments in overall satisfaction, and the results showed a gradual improvement in the score in the past years. Table 3 gives the subject-specific recognition results. Although the department had a small number of students in 2016 and 2019, the results indicated gradual improvement.

**Table 3:** Survey questionnaire for subjective recognition by school

Survey contents	2020 (n = 109)		2019 (n = 21)		2018 (n = 29)		2017 (n = 33)		2016 (n = 56)	
	Avg	SD	Avg	SD	Avg	SD	Avg	SD	Avg	SD
1. I was aware of the objectives and took part in the practice	4.51	0.60	4.15	0.81	4.17	0.54	3.94	0.80	4.02	0.82
2. The clinical practice was conducted as planned in advance	4.65	0.53	4.24	0.77	4.14	0.79	4.00	0.90	4.16	0.71
3. The content was appropriate at the student level	4.67	0.53	4.14	0.85	4.10	0.86	4.00	0.94	4.07	0.89
4. Clinical practice proceeded without wasting time	4.61	0.62	4.19	0.81	3.93	0.88	3.97	0.81	4.00	1.03
5. There was a chance to encounter patients with various diseases	4.54	0.66	4.14	0.85	3.93	1.00	3.85	0.91	3.73	1.04
6. There was ample opportunity for a checkup or a skill test	4.36	0.82	4.19	0.81	3.62	1.18	3.67	1.02	3.54	1.16
7. The professors were interested in student education	4.70	0.52	4.24	0.83	4.38	0.68	4.18	0.68	4.18	0.79
8. Fellows and residents were interested in student education	4.65	0.55	4.29	0.78	4.24	0.79	4.06	0.70	4.11	0.78
9. I received appropriate feedback on the clinical practice	4.64	0.59	4.25	0.85	4.10	0.77	4.06	0.79	4.02	0.86
10. I was judged on what I practiced rather than on knowledge	4.55	0.76	4.30	0.80	4.03	0.91	4.06	0.79	3.82	0.96
11. The criteria for re-training clinical practice were appropriate	4.55	0.71	4.25	0.85	4.29	0.71	4.06	0.70	–	–
12. The overall course was generally satisfactory	4.68	0.52	4.29	0.78	4.21	0.77	4.06	0.75	4.04	0.85
Overall satisfaction average	4.59	–	4.22	–	4.10	–	3.99	–	3.97	–

**Fig. 6:**
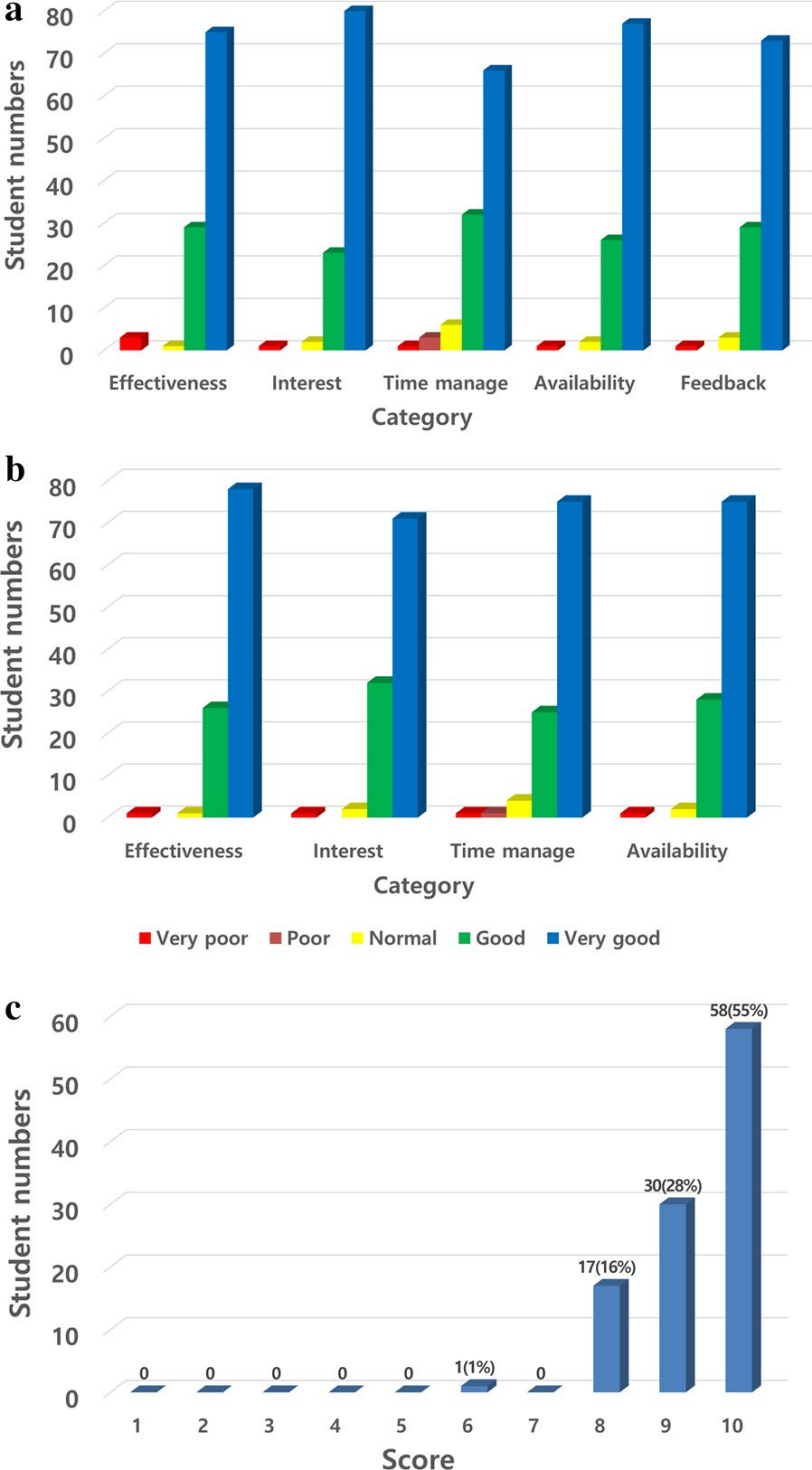
Results of the online survey by our own department. **A** Instructor evaluation. **B** Website and video lecture evaluation. **C** Overall satisfaction

Figure 6 shows the results for the online questionnaire survey conducted by our department. For instructor evaluation, 69%, 75%, 61%, 73%, and 69% of the students responded “very good” to the items on effectiveness, ability to draw interest, time management, availability, and feedback, respectively. For the website and video lecture evaluation, 74%, 67%, 71%, and 71% of the students rated as “very good,” their effectiveness, ability to draw interest, time management, and availability respectively. The average overall satisfaction score was 9.36, and 55% of the students chose 10 (best).

## Discussion

In the present study, we described the implementation and evaluation of a flipped learning model in an undergraduate medical program. The world has fundamentally changed since the onset of the COVID-19 pandemic in 2020 [4]. Due to the COVID-19 pandemic, healthcare professionals experienced difficulties due to situational and organizational factors, such as a higher workload, psychological stress, and a shortage of personal protective equipment [5]. Despite these difficulties, automation in radiotherapy delivery [6], and the development of new digital and virtual reality systems seem to have solved the problems caused by the COVID-19 pandemic effectively. Advances in technology have resulted in a variety of changes in medicine, including in the relationships between patients and doctors, and between teachers and students. Because of the COVID-19 crisis, the traditional mode of education that valued face-to-face interaction, quickly transitioned to a hybrid type that adopted virtual reality (VR) and flipped learning. Our study proved that this new approach reduced the burden of the Radiation Oncology Department during COVID-19 pandemic without reducing education efficacy and student’s enthusiasm toward education.

Virtual reality, which had been rapidly evolving in the areas of gaming and education, has emerged as an innovative media technology in the medical service software market in conjunction with advances in mobile and display technologies. VR has been applied in education related to surgical instruments, dental techniques using Haptic technology, surgical contents, and experience in the operating room [7, 8]. Bekelis et al. reported that patients who experienced preoperative VR had increased satisfaction with the surgical procedure [9]. The radiotherapy workflow is a complex multi-step process that is not easily understood by patients and medical students. For this reason, we have conducted and completed the accrual of the prospective randomized study to investigate the clinical usefulness of VR in patients who underwent breast RT (NCT04141943).

Another radical change in medicine is the more frequent application of artificial intelligence (AI) and deep learning (DL). Cancer detection and automatic segmentation using AI and DL are emerging research topics. For example, Xue et al. reported the performance outcomes of 3D convolution network in the detection of brain metastasis [10]; Tang et al. suggested that automatic segmentation using DL could delineate the volume of the brain glioma for radiotherapy [11]. Thus, AI and DL can replace much of what doctors and health care providers used to do.

Education has also been revolutionized by technological advancements. A systematic review of 82 papers concluded that flipped learning is a promising approach to increasing student motivation and engagement; however, the evidence for its effectiveness with respect to knowledge retention and transfer remains lacking [12]. Quantitative and qualitative feedback by students on flipped learning has been highly positive. Specifically for medical students’ education, flipped learning has shown significant improvement in student satisfaction compared with conventional lectures, both in scale scores and free-text feedback [13, 14]. Flipped learning involves not only moving lectures outside the classroom but also proper design that ensures coherence between the face-to-face and online sections of the course. In our program for medical students, we focused on the coherence of the course components. Lessons from the lectures of the day were supplemented by in-person activities, such as site visits or target contouring sessions.

Telemedicine is defined as “the remote diagnosis and treatment of patients by means of telecommunications technology” and can improve patients’ health with reduced costs. In the era of COVID-19 pandemic, the World Health Organization highlighted telemedicine as an essential service [15]. Historically, telemedicine is the most effectively integrated part of the medical school’s clerkship curriculum [16]. Although the Korean government is not legally permissible to telemedicine, it is actively attempting in making the rounds and medical student’s education. Our study, therefore, can be seen as a preparatory stage for the implementation of telemedicine, and in the process, flipped learning was used.

Nonetheless, flipped learning has some shortcomings. Because of the lack of feedback, some lectures could not accomplish their purpose of providing the appropriate knowledge needed by the student. Students also have fewer interactions with their instructors, who are required to spend a considerable amount of time preparing video lectures. These lectures may also become outdated and require frequent updating, considering the advances in therapy. Thus, instructors will be required to allocate much of their time to prepare and update the lecture videos. Many education specialists insist that face-to-face communication is indispensable in education. However, under special circumstances such as the COVID-19 pandemic, these traditional modes need to be replaced. Innovations in media and technology therefore need to be leveraged to address communication issues.

Our study was limited by the short duration and small sample. However, we intend to use the same program in 2021, and we look forward to being evaluated by the students. We also recognize the non-response bias of the students, which may have had an impact on the feedback results. In addition, since there was no opportunity to gain actual experience with patients who were treated with radiotherapy, the fact that the students did not have the opportunity to experience outpatient follow-up or toxicity management after radiation therapy, could be a major limitation for them. Another limitation was that the authors were all participants in the delivery of the flipped learning module. Evaluation undertaken by researchers not involved in the delivery may reduce such bias.

After the COVID-19 pandemic ends, a new educational program will be required. It is an essential part of a student’s program to perform physical examinations and history-taking in person with a patient, which had been suspended due to COVID-19. At the same time, flipped learning, based on our experience of the pandemic period, will also be used.

## Conclusions

In conclusion, disruption to medical education due to the COVID-19 crisis was avoided with the use of online contents and flipped learning. In the future “new normal,” minimized face-to-face interactions, such as flipped learning, may need to be actively utilized for medical and other students’ education, to accommodate various needs and circumstances of students.

## Supplementary Information

**Additional file 1: Figure 1** Online questionnaire for students’ customized schedule.

**Additional file 2: Figure 2** Online questionnaire for post-program survey.

## Data Availability

The datasets used and/or analyzed during the current study are available from the corresponding author on reasonable request.

## Abbreviations

CT: Computed tomography
VR: Virtual reality
AI: Artificial intelligence
DL: Deep learning
