# Supplementary figures and images for: Medical student education through flipped learning and virtual rotations in radiation oncology during the COVID-19 pandemic: a cross sectional research
Source: Radiat Oncol. 2021 Oct 16;16:204. doi: 10.1186/s13014-021-01927-x (PMC8520071; doi:10.1186/s13014-021-01927-x)

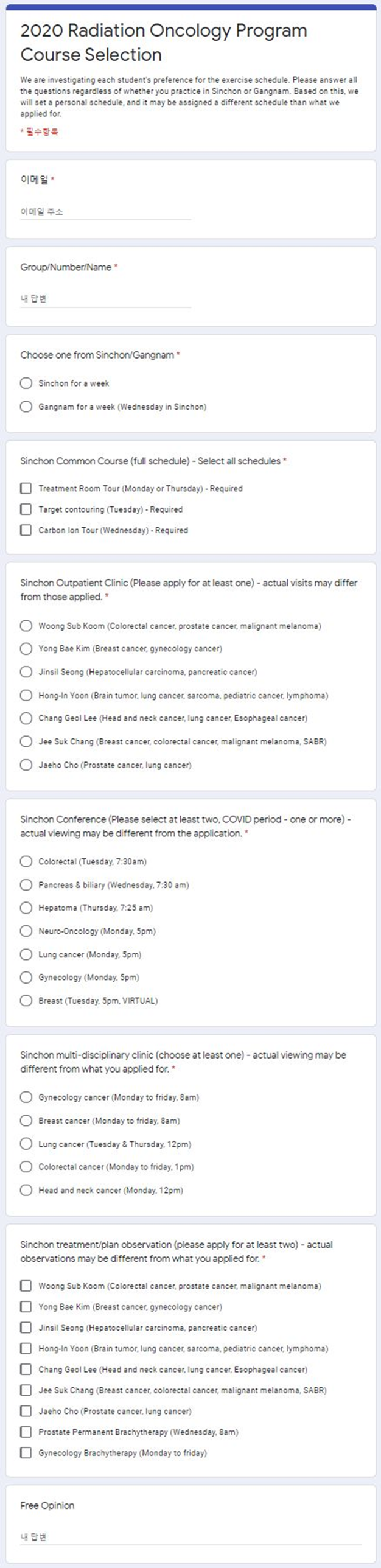

Supplement: Supplementary file 1 — Additional file 1: Figure 1 Online questionnaire for students’ customized schedule. [file 13014_2021_1927_MOESM1_ESM.tif]

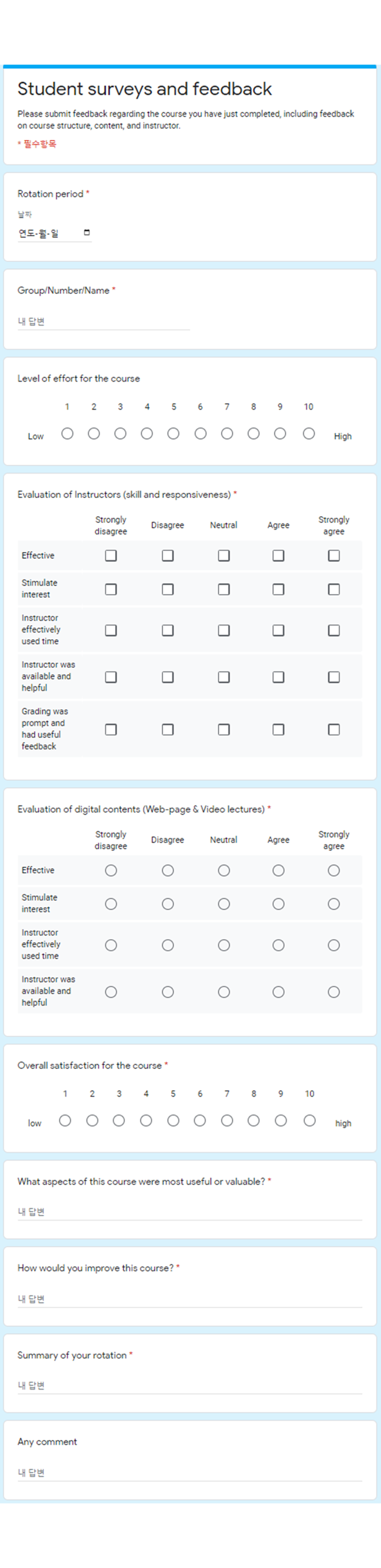

Supplement: Supplementary file 2 — Additional file 2: Figure 2 Online questionnaire for post-program survey. [file 13014_2021_1927_MOESM2_ESM.tif]
